# Percutaneous endoscopic transforaminal vs. interlaminar discectomy for L5–S1 lumbar disc herniation: a retrospective propensity score matching study

**DOI:** 10.1186/s13018-024-04543-z

**Published:** 2024-01-13

**Authors:** Tusheng Li, Guangnan Yang, Wei Zhong, Jiang Liu, Zhili Ding, Yu Ding

**Affiliations:** 1 Orthopedics of TCM Senior Department, The Sixth Medical Center of PLA General Hospital, 6 Fucheng Road, Haidian District, Beijing, 100048 People’s Republic of China; 2 The Second School of Clinical Medicine, Southern Medical University, Guangzhou, People’s Republic of China; 3 Department of Orthopaedics, School of Medicine, South China University of Technology, Guangzhou, People’s Republic of China; 4 Navy Clinical College, The Fifth School of Clinical Medicine, Anhui Medical University, Hefei, People’s Republic of China

**Keywords:** Lumbar disc herniation, Percutaneous endoscopic lumbar discectomy, Interlaminar approach, Transforaminal approach, Propensity score matching

## Abstract

**Objective:**

Percutaneous endoscopic lumbar discectomy (PELD) is a safe and effective minimally invasive surgery for treating lumbar disc herniation (LDH); however, the comparative clinical efficacy of percutaneous endoscopic transforaminal discectomy (PETD) and percutaneous endoscopic interlaminar discectomy (PEID) in treating L5–S1 LDH remains unclear. This study compared the clinical advantages of PEID and PETD for treating L5–S1 LDH.

**Methods:**

This was a single-centre retrospective study analysing clinical data from 120 patients with L5–S1 LDH between February 2016 and May 2020. Propensity score matching (PSM) was used to adjust for imbalanced confounding variables between the two groups. Perioperative data were recorded, and clinical outcomes, including functional scores and imaging data, were compared between groups. Functional scores included visual analogue scale (VAS) for back and leg pain, Oswestry disability index (ODI), and modified MacNab criteria. Imaging data included disc height index (DHI), ratio of greyscale (RVG), and range of motion (ROM) of the responsible segment.

**Results:**

After PSM, 78 patients were included in the study, and all covariates were well balanced between the two groups. In the matched patients, the PEID group showed significantly shorter surgical time (65.41 ± 5.05 vs. 84.08 ± 5.12 min) and lower frequency of fluoroscopy (2.93 ± 0.63 vs. 11.56 ± 1.54) compared with the PETD group (*P* < 0.001). There were no statistically significant differences in intraoperative blood loss, postoperative hospital stay, total incision length, and incidence of complications between the two groups (*P* > 0.05). After surgery, both groups showed significant improvement in back and leg pain based on VAS and ODI scores (*P* < 0.05). There were no statistically significant differences in clinical functional scores and imaging data between the two groups at various time points after surgery (*P* > 0.05). According to the modified MacNab criteria, the excellent and good rates in the PEID group and PETD group were 91.89% and 89.19%, respectively, with no statistically significant difference (*P* > 0.05).

**Conclusion:**

PEID and PETD have similar clinical efficacy in treating L5–S1 disc herniation. However, PEID is superior to PETD in reducing operation time and frequency of fluoroscopy.

## Introduction

Lumbar disc herniation (LDH) is a common and prevalent spinal disease with an increasing incidence, affecting 1–5% of the global population annually [1]. Approximately 80% of Americans have been reported to experience at least one episode of LDH and subsequent low back pain in their lifetime [2]. Conservative treatment is the first-choice approach for patients with LDH, but some patients need surgical intervention after conservative treatment to alleviate pain [3]. With the popularization of the minimally invasive concept and the innovation in the design of surgical instruments, minimally invasive spine endoscopy has become the mainstream surgical technique for treating LDH due to its advantages, such as minimal trauma, rapid recovery, and shorter hospitalization [4, 5]. Currently, percutaneous endoscopic lumbar discectomy (PELD) is widely used in the clinical treatment of LDH and has achieved favourable clinical outcomes [4]. PELD includes two different surgical approaches, namely percutaneous endoscopic transforaminal discectomy (PETD) and percutaneous endoscopic interlaminar discectomy (PEID), each with its own advantages in treating LDH [6].

Compared with PEID, PETD removes the herniated disc through the "safety triangle" of the intervertebral foramina without laminectomy and dural retraction, causing less damage to the spinal canal and the soft tissues of the lumbar spine [7, 8]. Some researchers believe that PETD can more effectively relieve postoperative pain and reduce blood loss and hospital stay [8]. However, for L5–S1 disc herniation, PETD still faces significant technical challenges due to its challenging anatomical characteristics such as a high position of iliac crest, narrow intervertebral foramen, and facet hypertrophy [6, 9]. On the other hand, PEID benefits from a wider interlaminar space and easier localization; thus, some scholars believe that it is a better choice for treating L5–S1 disc herniation and may facilitate surgery and shorten operative time [10]. Nevertheless, Yeung et al. [11] demonstrated that PETD can be successfully used in treating disc herniation at all lumbar levels, including L5–S1. Therefore, it is still controversial which surgical approach yields better outcomes in the treatment of L5–S1 disc herniation. In this study, we retrospectively analysed the clinical outcomes of patients with L5–S1 disc herniation who underwent two different surgical approaches. Our findings illuminate their comparative clinical safety and efficacy and provide some clinical rationale for selecting surgical methods.

## Materials and methods

### Study design and patients

This study was a single-centre retrospective cohort study approved by the Ethics Committee of the Sixth Medical Center of the PLA General Hospital (No. HZKY-PJ-2023–34). All patients provided written informed consent before treatment. From February 2016 to May 2020, 120 patients with L5–S1 disc herniation who underwent PEID or PETD were included in this study according to inclusion and exclusion criteria (Table 1). Among them, 63 patients underwent PEID, and 57 patients underwent PETD. Due to the inherent imbalances in covariates between the two groups, we adopted propensity score matching (PSM) (matching tolerance set at 0.02) to balance the influence of confounding factors when comparing clinical outcomes. The propensity score for each patient was calculated as the probability of accepting different surgical treatments, including all covariates considered clinically relevant and possibly affecting clinical outcomes. The following variables were used for PSM: (1) age; (2) body mass index (BMI); (3) gender; (4) disease duration; (5) smoking history; (6) medical history; (7) pathological classification; and (8) follow-up time.

**Table 1:** Inclusion and exclusion criteria

Inclusion criteria	Central, paracentral, or prolapsed L5–S1 disc herniation Symptoms of low back pain and leg pain Failure of formal conservative treatment
Exclusion criteria	Lumbar instability, such as lumbar spondylolisthesis Intervertebral disc inflammation or tuberculosis Severe lumbar stenosis, and far lateral disc herniation Previous surgery at the lumbar spine Multiple segments of disc herniation Recurrent disc herniation Pregnant or syndrome of cauda equina Severe cardiac disease, active neoplasm, anaemia, or any other surgical contraindications

### Surgical methods

#### PEID approach

**Fig. 1:**
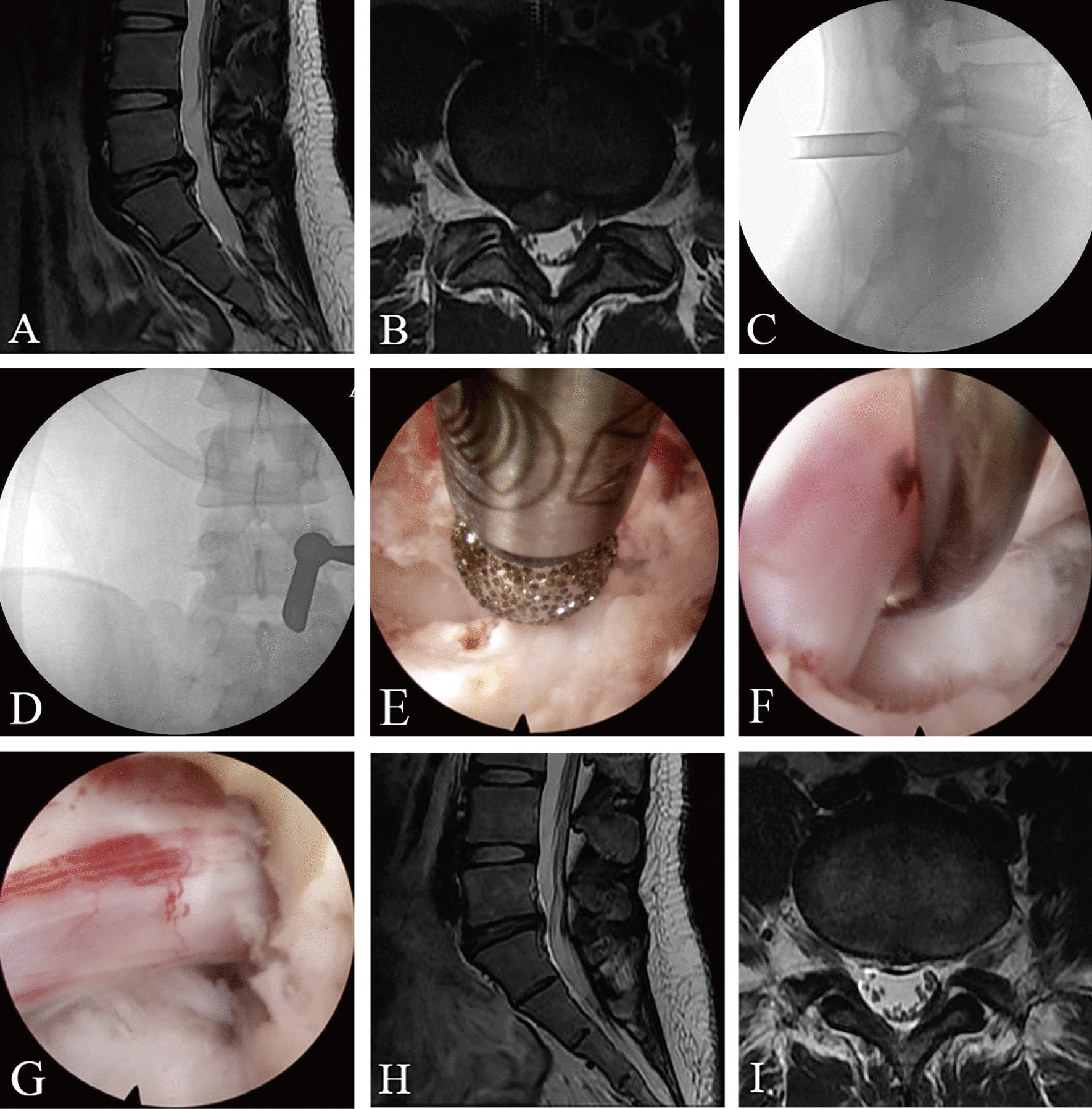
Images from a patient treated with PEID. **A** and **B** Preoperative MRI showed L5–S1 disc herniation. **C** and **D** The position of the working cannula through the inter lamina approach during the operation. **E** Intraoperative grinding drill for laminoplasty. **F** Endoscopic demonstration of disc herniation with nerve root compression. **G** Nerve root decompression. **H** and **I** Postoperative MRI showed that the L5–S1 herniated intervertebral disc has been removed

Patients in the PEID group were placed in a prone position under basic combined local anaesthesia. The puncture point of the body surface projection of the responsible segment was identified under C-arm X-ray fluoroscopy, which was located 1–2 cm lateral to the midline. Then, a puncture needle was inserted and the tip landing point was confirmed to be positioned within the interlaminar space and adjacent to the medial margin of the facet joint. A 7–10 mm surgical incision was made, and a soft tissue dilator was used to expand the surgical access. Next, the working cannula was placed and the endoscopic system was installed. After the endoscopic resection of part of the superior lamina, inferior lamina, and ligamentum flavum, epidural fat, nerve roots, and dural sac were clearly exposed. The herniated disc was excised at the lateral shoulder or medial axilla of the nerve root according to the location of the herniated disc or the patient's reaction to the radicular pain during the operation. The nerve root was gently retracted using a nerve dissector to expose the herniated disc. Thereafter, the herniated disc was removed using disc forceps under endoscopic visualization, and the endoscope angle was adjusted to explore the nerve root and confirm complete removal. Decompression was considered successful if the nerve root tension decreased, no compressive tissue remained around the nerve, and the nerve root and dura sac showed autonomous pulsation. After completing decompression, the operative area was confirmed to have no active bleeding, and the endoscope and working cannula were removed. Representative cases are shown in Fig. 1.

#### PETD approach

**Fig. 2:**
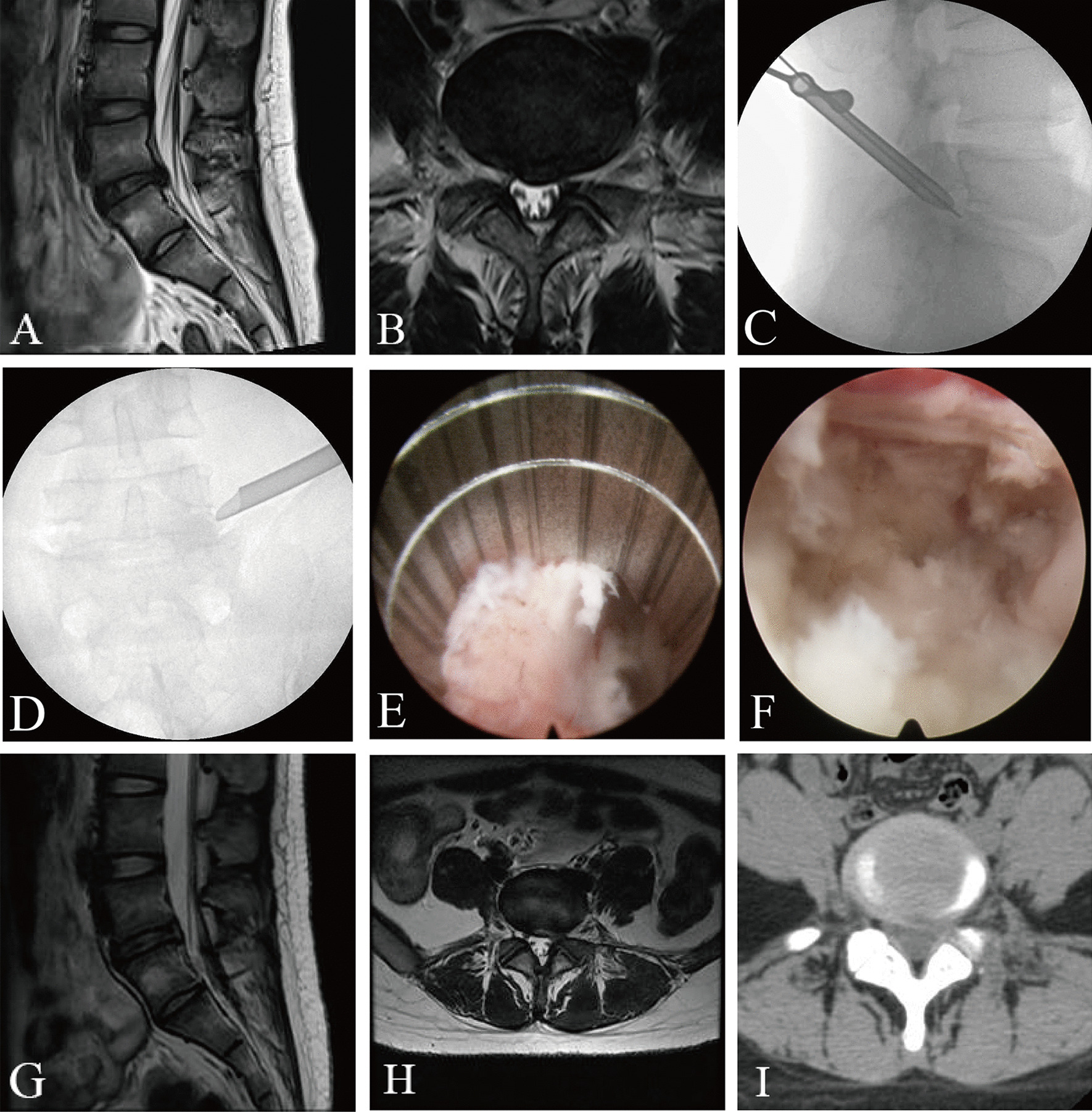
Images from a patient treated with PETD. **A** and **B** Preoperative MRI showed L5–S1 disc herniation. **C** and **D** The position of the working cannula through the inter lamina approach during the operation. **E** Intraoperative ring sawing to remove part of facet joints.** F** Removal of herniated disc tissue. **G** to **I** Postoperative MRI and CT showed that the L5–S1 herniated intervertebral disc has been removed

Similar to PEID, patients in the PETD group were also placed in the prone position under the same protocol of anaesthesia. The responsible segment was identified using C-arm fluoroscopy, and the puncture point was located 12–15 cm lateral to the midline. The puncture needle was inserted at an angle of 15–25° to the horizontal plane. Its correct position was confirmed under fluoroscopy if: (1) the anteroposterior view showed that the needle tip intersected the inner margin of the vertebral pedicle and (2) the lateral view showed that the needle tip was located above the posterior margin of the intervertebral disc. Then, a 7–10 mm surgical incision was made, and a soft tissue dilator was used to expand the surgical access. If necessary, intraoperative foraminal enlargement with a ring saw was performed to remove part of the hypertrophic bone and facet joints. A working cannula was inserted, and the endoscopic system was installed. Under endoscopic visualization, the herniated disc was completely exposed, and the protruded material was removed using disc forceps. Thereafter, the endoscope was adjusted to explore and release the nerve root. After completing decompression, the operative area was observed to confirm the absence of active bleeding. Finally, the endoscope and working cannula were removed, followed by the closure of the working channel and skin suture. Representative cases are shown in Fig. 2.

### Data collection and measurements

Baseline data and perioperative data of all successfully matched patients with L5–S1 disc herniation were collected. Perioperative data included operation time, frequency of fluoroscopy, intraoperative blood loss, hospital stay, and total incision length. Regular follow-up was conducted at 3 months, 6 months, 12 months postoperatively, and the last follow-up through phone calls and/or emails to record patients' clinical functional scores, imaging data, and incidence of complications.

### Clinical assessment

Clinical functional scores were determined using self-assessment questionnaires, including visual analogue scale (VAS) for back and leg pain, Oswestry disability index (ODI), and modified MacNab criteria. Furthermore, we used minimal clinically important difference (MCID) to assess the clinical significance of changes in VAS and ODI. An MCID value of a change of 2 or greater for VAS and 13 or greater for ODI was considered clinically significant [12, 13]. At the last follow-up, patients’ satisfaction was assessed using the modified MacNab criteria as follows: excellent: complete disappearance of symptoms with the ability to resume original work and daily activities; good: mild symptoms with slight restriction of activities but no impact on work and daily life; fair: symptom relief with moderate restriction of activities and impact on normal work and daily life; poor: no improvement or even worsening of symptoms [14]. The excellent and good rates were calculated as (excellent + good) / total number of cases *100%.

### Imaging measurements

Patients in both groups underwent X-ray imaging of the lumbar spine in the lateral, flexion, and extension positions. In addition, magnetic resonance imaging (MRI) was performed. Imaging data were collected using Image Viewer or AnyPacs software installed on workstations in DICOM or JPG format. All imaging data were measured three times by three independent evaluators, and the average values were collected.

1. Disc height index (DHI) was used to assess changes in disc height at different follow-up time points, as previously described [15]. The anterior, middle, and posterior heights of the upper and lower vertebral bodies and discs were measured on lateral lumbar spine X-ray. DHI was calculated as the ratio of the sum of intervertebral disc heights to the sum of upper and lower vertebral body heights: DHI = 2(b1 + b2 + b3) / (a1 + a2 + a3 + c1 + c2 + c3) * 100% (Fig. 3A).

**Fig. 3:**
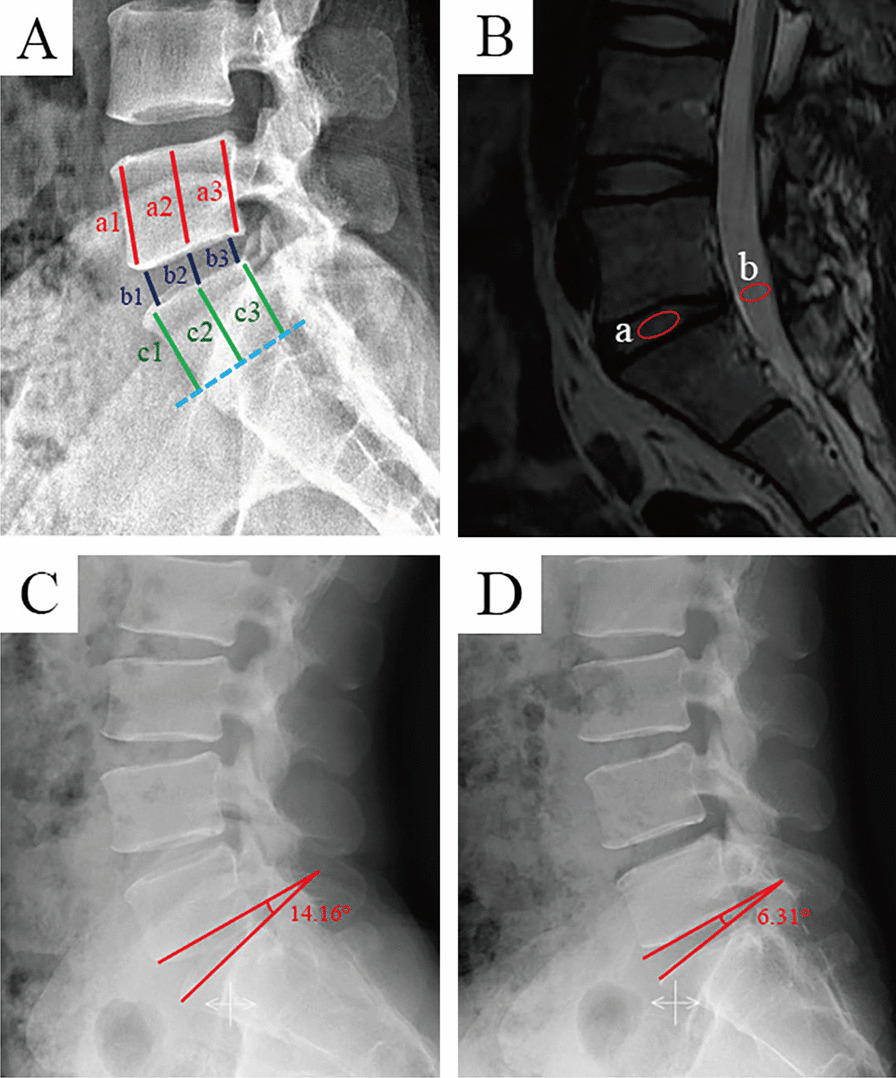
Schematic of imaging measurements. **A** Disc height index (DHI), DHI = [2(b1 + b2 + b3)] / [(a1 + a2 + a2) + (c1 + c2 + c3)] *100%. **B** Ratio value of the greyscale (RVG). Midsagittal T2-weighted images were chosen, and RVG was the greyscale of discs a normalized against the greyscale of cerebrospinal fluid at the same level b. **C** and **D** Schematic diagram of the range of motion (ROM). **C** the segmental angle in hyperextension position (14.16°). **D** the segmental angle in hyperflexion position (6.31°); the range of motion of the segment (ROM = 14.16 − 6.31° = 7.85°)
2. The ratio value of the greyscale (RVG) was measured based on the modified Schneiderman method to evaluate disc hydration [16]. The MRI midsagittal T2-weighted images were imported into Photoshop software (Adobe Photoshop 2023 version), and the average grayscale value of the intervertebral disc and cerebrospinal fluid were measured at the same segment. RVG was calculated as the ratio of the average grayscale value of the intervertebral disc to the average grayscale value of cerebrospinal fluid: RVG = (average grayscale value of the intervertebral disc / average grayscale value of cerebrospinal fluid) * 100% (Fig. 3B).
3. Range of motion (ROM) was measured on lumbar spine X-ray in hyperextension and flexion positions. The line connecting the inferior endplate of the superior vertebra to the superior endplate of the inferior vertebra formed an angle that was positive when measured posterior to the vertebra and negative when measured anterior to the vertebra. The difference between the angles in hyperextension and flexion was considered a segmental range of motion (ROM = angle in hyperextension position—angle in hyperflexion position) (Fig. 3C and D). Based on the theory proposed by Frymoyer et al. [17], we concluded that lumbar instability exists in the L5–S1 segment at ROM > 20°.

### Statistical analysis

All statistical analyses were performed using SPSS version 25 (IBM SPSS Statistics for Windows, version 25.0. Armonk, NY: IBM Corp.). Student's *t*-test was used for continuous data that followed a normal distribution, and the results are expressed as mean ± SD. Within-group comparisons at different time points were analysed using repeated measures analysis of variance (ANOVA). The nonparametric test was used for data without normal distribution. To assess the balance between the two groups, we calculated standardized mean differences (SMD) [13] to represent the intergroup balance of a given covariate. The SMD is not affected by sample size and compares the relative balance between variables [13]. According to the Cohen's criteria, an SMD ≤ 0.2 for a covariate indicates a small difference [18]. Categorical data were compared using the Chi-square test and are presented as frequency and percentage. A *p *value < 0.05 showed statistically significant intergroup differences.

## Results

### Baseline characteristics before and after PSM

In total, 120 patients with L5–S1 disc herniation were included in this study, with 63 cases in the PEID group and 57 cases in the PETD group, based on the inclusion and exclusion criteria. The baseline characteristics of the two groups before PSM are shown in Table 2. Covariates with SMD ≤ 0.2 and *P* > 0.05 were considered balanced and comparable between the two groups. However, we observed three covariates unbalanced between the two groups in Table 2, including age (SMD = 0.285, *P* = 0.120), disease duration (SMD = 0.542, *P* = 0.003), and follow-up time (SMD = 0.239, *P* = 0.212). After PSM, 78 patients with L5–S1 disc herniation were included in this study., The baseline characteristics of the two groups are shown in Table 3, indicating that all covariates between the two groups were balanced and comparable.

**Table 2:** Baseline characteristics before propensity score matching

Demographics	PEID group (*n* = 63)	PETD group (*n* = 57)	SMD	*P* value
Age (years)	41.76 ± 11.00	44.84 ± 10.47	**0.285**	0.120
BMI (kg/m^2^)	24.84 ± 3.10	24.81 ± 3.03	0.010	0.959
Gender, *n* (%)			0.007	0.970
Male	40 (63.49)	36 (63.16)		
Female	23 (36.51)	21 (36.84)		
Medical history, *n* (%)				
Hypertension	32 (50.79)	30 (52.63)	0.037	0.841
Diabetes	27 (42.86)	25 (43.86)	0.020	0.912
Pathological classification, *n* (%)			0.038	0.823
Central	9 (14.29)	6 (10.53)		
Paracentral	31 (49.21)	29 (50.88)		
Prolapsus	23 (36.51)	22 (38.60)		
Disease duration (months)	23.22 ± 5.00	26.12 ± 5.35	**0.542**	**0.003**
Smoking, *n* (%)	29 (46.03)	26 (45.61)	0.008	0.963
Follow-up time	25.57 ± 1.50	25.93 ± 1.51	**0.239**	0.212

Bolding is to indicate SMD > 0.2 or P ≤ 0.05, which means that the corresponding confounders are not balanced between the two groups

**Table 3:** Baseline characteristics after propensity score matching

Demographics	PEID group (*n* = 39)	PETD group (*n* = 39)	SMD	*P* value
Age (years)	45.05 ± 10.07	44.51 ± 10.69	0.052	0.819
BMI (kg/m^2^)	24.69 ± 2.99	24.81 ± 2.90	0.041	0.867
Gender, *n* (%)			0.055	0.808
Male	27 (69.23)	26 (66.67)		
Female	12 (30.77)	13 (33.33)		
Medical history, *n* (%)				
Hypertension	19 (48.72)	20 (51.28)	0.051	0.829
Diabetes	19 (48.72)	19 (48.72)	0.000	1.000
Pathological classification, *n* (%)			0.114	0.624
Central	5 (12.82)	5 (12.82)		
Paracentral	16 (41.03)	20 (51.28)		
Prolapsus	18 (46.15)	14 (35.90)		
Disease duration (months)	24.97 ± 4.15	25.36 ± 5.78	0.078	0.629
Smoking, *n* (%)	21 (53.85)	21 (53.85)	0.000	1.000
Follow-up time	25.59 ± 1.48	25.67 ± 1.51	0.054	0.848

### Perioperative data

All patients underwent surgery by the same team of surgeons. In the PEID group, the average operation time was 65.23 ± 4.95 min, frequency of fluoroscopy was 2.97 ± 0.63, intraoperative blood loss was 35.13 ± 5.42 mL, hospital stay was 6.33 ± 0.96 days, and total incision length was 8.60 ± 0.79 mm. In the PETD group, the average operation time was 85.31 ± 6.30 min, frequency of fluoroscopy was 11.38 ± 1.09, intraoperative blood loss was 36.10 ± 4.52 mL, hospital stay was 6.23 ± 0.87 days, and total incision length was 8.64 ± 0.81 mm. The PEID group had significantly shorter operation time and frequency of fluoroscopy compared with the PETD group (*P* < 0.001). However, there were no statistically significant differences between the two groups in terms of intraoperative blood loss, postoperative hospital stay, and total incision length (*P* > 0.05) (Table 4).

**Table 4:** Comparison of perioperative data between the two groups

	PEID group (*n* = 39)	PETD group (*n* = 39)	*P* value
Operative time (min)	65.23 ± 4.95	85.31 ± 6.30	< 0.001
Fluoroscopy times	2.97 ± 0.63	11.38 ± 1.09	< 0.001
Intraoperative blood loss (ml)	35.13 ± 5.42	34.10 ± 4.52	0.367
Hospital stay (*d*)	6.33 ± 0.96	6.10 ± 0.75	0.255
Total length of incision (cm)	8.60 ± 0.79	8.46 ± 0.88	0.539

### Clinical assessment

**Fig. 4:**
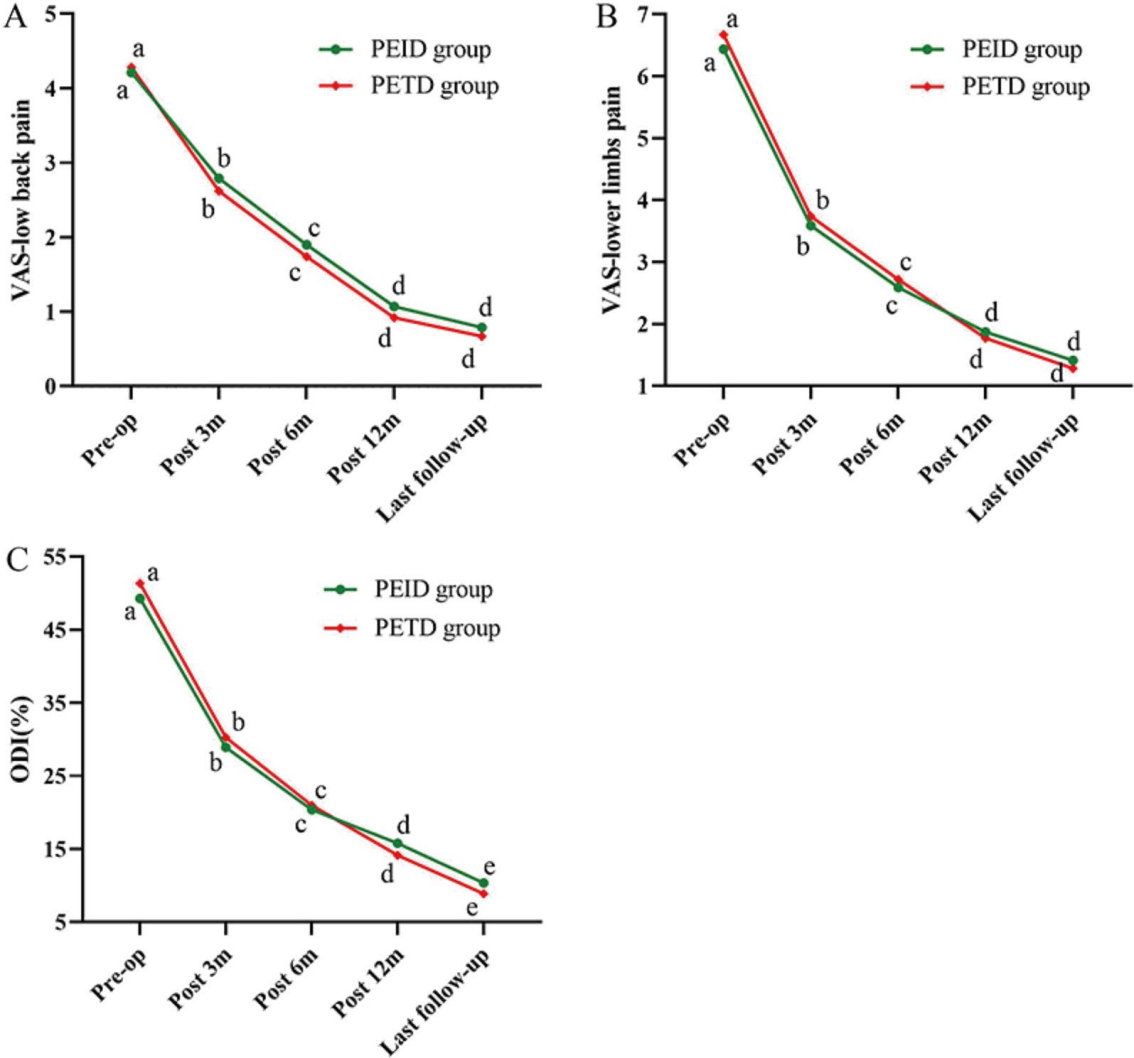
Results of clinical efficacy of functional scores. **A** Changes in VAS scores for low back pain over time. **B** Changes in VAS scores for lower limbs pain over time. **C** Changes in ODI over time. *VAS* visual analogue scale, *ODI* Oswestry disability index. a**–**e indicate the letter labelling of the time point difference (comparison within the group), if 2 time points have the same letter, there is no significant difference between the 2 time points (*P* > 0.05); otherwise, different letters at 2-time points mean the difference is significant (*P* ≤ 0.05)

The mean VAS scores for low back pain in the PEID and PETD groups decreased from 4.21 ± 0.89 and 4.28 ± 0.86 before the operation (*P* = 0.820) to 2.79 ± 0.98 and 2.62 ± 1.04 at 3 months postoperatively (*P* = 0.524), 1.90 ± 1.07 and 1.74 ± 0.99 at 6 months postoperatively (*P* = 0.644), 1.07 ± 1.01 and 0.92 ± 0.90 at 12 months postoperatively (*P* = 0.555), and 0.79 ± 0.92 and 0.67 ± 0.81 at the last follow-up (*P* = 0.610), respectively. Additionally, the mean VAS scores for lower limb pain in the PEID and PETD groups decreased from 6.44 ± 0.99 and 6.67 ± 0.93 before the operation (*P* = 0.315) to 3.59 ± 0.81 and 3.74 ± 0.88 at 3 months postoperatively (*P* = 0.402), 2.59 ± 0.85 and 2.72 ± 0.89 at 6 months postoperatively (*P* = 0.578), 1.87 ± 1.13 and 1.77 ± 0.84 at 12 months postoperatively (*P* = 0.854), and 1.41 ± 1.02 and 1.28 ± 0.89 at the last follow-up (*P* = 0.570), respectively. There were no statistically significant differences in VAS scores for low back pain and lower limbs pain between the two groups. Compared with before the surgery, both groups demonstrated significant improvement in VAS scores after the surgery (*P* < 0.001), and the improvement met the clinical significance criteria for MCID (Fig. 4A, B).

The mean ODI scores in the PEID and PETD groups decreased from 49.28 ± 9.36 and 51.33 ± 9.31 before the operation (*P* = 0.335) to 28.92 ± 7.41 and 30.26 ± 6.87 at 3 months postoperatively (*P* = 0.413), 20.36 ± 5.21 and 20.97 ± 5.71 at 6 months postoperatively (*P* = 0.620), 15.79 ± 5.44 and 14.15 ± 5.81 at 12 months postoperatively (*P* = 0.202), and 10.36 ± 6.49 and 8.87 ± 6.01 at the last follow-up (*P* = 0.297), respectively. The difference in ODI between the two groups was not statistically significant. Compared with those before the operation, the postoperative ODI significantly improved (*P* < 0.001) in both groups, which also met the clinical significance criteria for MCID (Fig. 4C).

At the last follow-up, according to the modified MacNab criteria, 25 cases were rated as excellent, 11 cases as good, 3 cases as fair, and 0 cases as poor in the PEID group, with an excellent and good rate of 92.30%. In the PETD group, 23 cases were rated as excellent, 12 cases as good, 3 cases as fair, and 1 case as poor, with an excellent and good rate of 89.74%. There was no significant difference in the excellent and good rates between the two groups (*P* = 0.771) (Table 5).

**Table 5:** Comparison of MacNab evaluation and complications between the two groups

	PEID group (*n* = 39)	PETD group (*n* = 39)	*P* value
MacNab evaluation			0.771
Excellence	25	23	
Good	11	12	
Fair	3	3	
Poor	0	1	
Excellence/good rate	92.30%	89.74%	
Complications			1.000
Low back pain or lower limbs pain	2	1	
Recurrent disc herniation	0	1	
Dural sac tear	0	0	
Disc space infection	0	0	

During the follow-up period, 4 patients experienced complications with a complication rate of 5.13%. Two patients in the PEID group and one patient in the PETD group experienced worsening of lower limb neurological symptoms postoperatively. In the PETD group, one patient experienced the recurrence of LDH after surgery. However, there was no significant difference in the complication rates between the two groups (*P* = 1.000) (Table 5). No serious complications such as dural tear or disc space infection, were detected among all patients.

### Image measurement

**Fig. 5:**
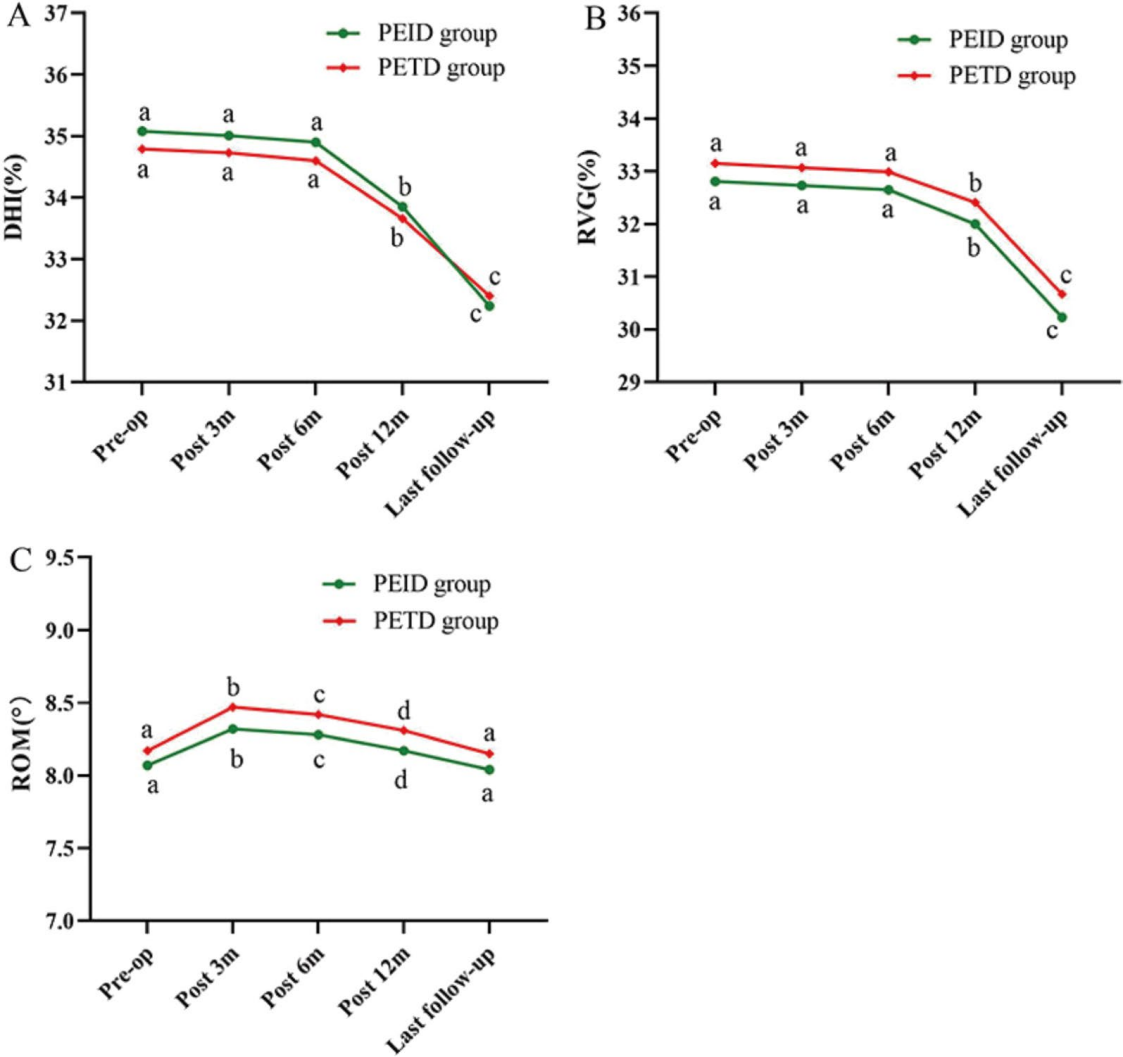
Results of imaging measurement. **A** Changes in DHI during the follow-up.** B** Changes in RVG during the follow-up. **C** Changes in ROM during the follow-up. DHI, Disc Height Index; RVG, the ratio value of the greyscale; ROM, the range of motion; a-d indicate the letter labelling of the time point difference (comparison within the group); if two time points have the same letter, there is no significant difference between the two time points (*P* > 0.05); otherwise, different letters at 2-time points mean the difference is significant (*P* ≤ 0.05)

In both groups, the average DHI of the responsible segments showed a decreasing trend (Fig. 5A). The mean DHI in the PEID and PETD groups decreased from (35.08 ± 2.74)% and (34.79 ± 2.77)% before the operation (*P* = 0.646) to (35.01 ± 2.67)% and (34.73 ± 2.75)% 3 months postoperatively (*P* = 0.642), (34.90 ± 2.55)% and (34.60 ± 2.69)% 6 months postoperatively (*P* = 0.619), (33.85 ± 2.44)% and (33.66 ± 2.56)% 12 months postoperatively (*P* = 0.741), and (32.24 ± 2.31)% and (32.40 ± 2.51)% at last follow-up (*P* = 0.770), respectively. There was no statistically significant difference in DHI between the two groups. However, the difference in DHI at 12 months postoperatively and at the last follow-up was statistically significant when compared with the preoperative period (*P* < 0.001) (Fig. 5A).

The mean RVG values in the PEID and PETD groups decreased from (32.81 ± 2.78)% and (33.15 ± 2.72)% before the operation (*P* = 0.590) to (32.73 ± 2.76)% and (33.07 ± 2.64)% 3 months postoperatively (*P* = 0.579), (32.65 ± 2.70)% and (32.99 ± 2.59)% 6 months postoperatively (*P* = 0.565), (32.00 ± 2.55)% and (32.41 ± 2.46)% 12 months postoperatively (*P* = 0.477), and (30.23 ± 2.51)% and (30.67 ± 2.41)% at last follow-up (*P* = 0.432), respectively. The trends in RVG values were similar between the two groups, with no statistically significant difference. Compared with baseline, RVG values showed a statistically significant difference at 12 months postoperatively and at the last follow-up (*P* < 0.001) (Fig. 5B).

The mean ROM values in the PEID and PETD groups were (8.07 ± 0.46)° and (8.17 ± 0.49)° before the operation (*P* = 0.347), (8.32 ± 0.45)° and (8.47 ± 0.48)° at 3 months postoperatively (*P* = 0.150), (8.28 ± 0.44)° and (8.42 ± 0.45)° at 6 months postoperatively (*P* = 0.174), (8.17 ± 0.40)° and (8.31 ± 0.42)° at 12 months postoperatively (*P* = 0.155), and (8.04 ± 0.36)° and (8.15 ± 0.38)° at the last follow-up (*P* = 0.186), respectively. There was no statistically significant difference in ROM values between the two groups. The ROM values increased after surgery in both groups, but the ROM values were all less than 20°, indicating no cases of lumbar instability in either group (Fig. 5C).

## Discussion

LDH is a chronic progressive disease that clinically presents with low back pain, radicular symptoms in the lower limbs, and sensory disturbances in the corresponding dermatomes [19]. Surgical intervention is required for patients with LDH who do not respond to conservative treatment. [3, 20]. Traditional open surgery is a classic approach for treating LDH but necessitates it involves extensive dissection of the paraspinal muscles and wide removal of the lamina, and facet joints, which can lead to postoperative complications such as refractory low back pain, muscle denervation, and lumbar instability [7, 21]. With the advancement of endoscopy techniques, several studies have shown that endoscopic treatment of LDH can achieve similar clinical outcomes as open surgery, with advantages such as less trauma, faster recovery, and fewer complications [4, 5]. Currently, PEID and PETD are widely used in the treatment of patients with LDH, with specific clinical characteristics [22]. However, for L5–S1 disc herniation, the specific advantages and disadvantages of these two different surgical approaches are still unclear due to their unique anatomical characteristics.

During the endoscopic treatment of L5–S1 disc herniation, the presence of the high iliac crest and a narrow intervertebral foramen can hinder the entry of the working cannula, and the hyperplastic facet joints can also obstruct the protruding disc [6]. The lateral approach has a limited perforation angle, necessitating excessive intraoperative removal of facet joints that compromise the biomechanical stability of the spine. These limitations increase the difficulty of intraoperative accurate puncture positioning and adequate surgical decompression for PETD, necessitate repeated fluoroscopy, and increase radiation exposure for both patients and spine surgeons [9]. Excessive X-ray exposure is a serious concern and can have significant health implications for medical personnel in the long term [23]. In addition, due to the obstruction caused by the superior margin of the iliac crest and the narrow intervertebral foramen, the operating space during PETD is insufficient, which may lead to incomplete removal of the protruding disc in the far region. The residual disc tissue increases the risk of recurrent disc herniation, which can seriously affect the treatment outcome [6]. Therefore, it is usually recommended to remove the L5–S1 disc through a wide interlaminar space during PEID. In the L5–S1 segment, the sacral one nerve root originating from the dural sac is positioned high in the plane of the intervertebral spaces, The herniated disc tissue can be removed intraoperatively from the shoulder or axilla of the nerve root according to the actual situation, which is easier and safer in this segment than in other segments. However, PEID necessitates the removal of the ligamentum flavum and a part of the lamina, which may interfere with the dural sac and increase the risk of injury to the dural sac or cauda equina [24]. Therefore, caution is needed during PEID to reduce the incidence of postoperative complications.

In our study, we observed that both PEID and PETD for L5–S1 disc herniation showed no significant difference in clinical functional scores and imaging. VAS scores for back and leg pain and ODI index of all patients significantly improved after the surgery, meeting the criteria for MCID. Therefore, both minimally invasive surgical techniques are safe and effective treatments for L5–S1 disc herniation, leading to favourable clinical outcomes. However, despite similar clinical outcomes of the two surgical approaches, they still have distinct characteristics in treating L5–S1 herniated discs. We found that the PEID group had significantly shorter operation time and lower frequency of fluoroscopy than the PETD group, suggesting the superiority of PEID over PETD in reducing surgical time and radiation exposure. The advantages of PEID may be related to several factors: (i) PEID uses an interlaminar approach, which is familiar to most spine surgeons and easier compared with PETD; (ii) PEID is not technically limited by the obstruction of the high iliac crest and narrow intervertebral foramen, allowing rapid and precise puncture positioning, and easy targeting of the L5–S1 herniated disc within a wide interlaminar space; (iii) PEID provides a spacious operating space, allowing better mobility of the working cannula for complete removal of the protruding disc; and (iv) PEID directly visualizes the protruded or extruded disc under endoscopy, enabling full decompression of central and paracentral disc herniation [6].

Although PETD for L5–S1 disc herniation is more challenging and requires higher proficiency of spine surgeons, it has its advantages and indications. PETD demands entering the spinal canal through a physiologically formed safe triangle of the intervertebral foramen, avoiding the blockade of the dural sac and nerve root traction during the surgery. Thus, PETD can reduce the incidence of complications, such as dural tear and nerve root injury. Besides, PETD is a better choice for patients with recurrent LDH as it can effectively avoid the influence of scar tissue from previous surgeries through the intervertebral foramen approach [10]. PETD can treat almost all types of L5–S1 disc herniations, including central, paracentral, foraminal, and far lateral types. However, giant herniated discs are relative contraindications for PETD, primarily due to limited surgical space caused by the obstruction of the superior margin of the iliac crest and the narrow intervertebral foramen, which can lead to inadequate decompression of the herniated disc. On the other hand, PEID has advantages for central, paracentral, and freely isolated types of disc herniations since it is not limited by the iliac crest blockade and offers advantages such as rapid puncture positioning, shorter operation time, and lower frequency of fluoroscopy.

In our study, all patients were successfully operated under endoscopy without any severe complications, such as dural tears or disc space infections. Four patients experienced postoperative complications. Two patients in the PEID group and one patient in the PETD group suffered from aggravating radicular symptoms within three days after surgery. The symptoms of patients improved by nerve nutrition, hormone therapy, and rehabilitation. One patient in the PETD group experienced LDH recurrence at 12 months postoperatively, and the symptoms improved after conservative treatment. There was no statistically significant difference in the incidence of complications between the two groups (*P* > 0.05). In addition, the results of imaging revealed that the mobility of the responsible segment increased postoperatively compared with before surgery, implying that we should attempt to damage as little as possible of the original lumbar spine when decompressing the disc. Previous studies have shown that loss of structures such as facet joints, and intervertebral discs, can all affect lumbar spine biomechanics [25, 26]. In our study, both PETD and PEID were performed without intraoperative resection of the high-weight-bearing area of facet joints, which had less impact on segmental stability. After recovery from surgical trauma and lumbar functional exercises, the mobility of the responsible segment was restored, and no patient experienced segmental instability during follow-up. However, whether postoperative disc degeneration and decreased disc height affect patients' surgical prognosis needs further studies.

In summary, both PEID and PETD for L5–S1 disc herniation can achieve favourable clinical outcomes. However, PEID has advantages over PETD, with reduced operation time and fluoroscopy exposure. There are some limitations to the current study. First, it was a retrospective study, and we could not completely eliminate subjective factors in case selection during the study period. We also could not achieve random grouping. Although we used PSM to minimize confounding factors, some biases might still exist. Second, although the results of imaging were averaged after 3 measurements by 3 independent reviewers, measurement error might still exist. Thirdly, the sample size was relatively small, the follow-up period was short, and it was a single-centre study, which might influence the results.

## Data Availability

The datasets used during the current study are available from the corresponding author on reasonable request.
